# Polar and Tropical Worlds

**Published:** 1876-03

**Authors:** 


### Bibliographical.

The Polar and Tropical Worlds: A Description of Nature and Man in the Polar and Equatorial Regions of the Earth. By Drs. G. Hartwig and A. H. Guernsey. Published by E. Nebhut, Madison, Ga.; pp. 811.

This work is designed to give a complete and full history of the travels and investigations of such distinguished explorers as Franklin, Hall, Kane, Agassiz and others. It treats largely of the climate of both the polar and tropical world; the changes taking place in temperature, and the influence which such changes have on man, both mentally and physically. The work is arranged in neat style, illustrated with over two hundred engravings, and would be a valuable addition both to the libraries of the literary and scientific.

